# Aberrant Interhemispheric Connectivity in Obstructive Sleep Apnea–Hypopnea Syndrome

**DOI:** 10.3389/fneur.2018.00314

**Published:** 2018-05-08

**Authors:** Yu-Ting Liu, Hui-Xin Zhang, Hui-Jun Li, Ting Chen, Ya-Qing Huang, Lian Zhang, Zhi-Chun Huang, Bin Liu, Ming Yang

**Affiliations:** ^1^Department of Radiology, Children’s Hospital of Nanjing Medical University, Nanjing, China; ^2^School of Medicine, Southeast University, Nanjing, China; ^3^Department of Otolaryngology-Head and Neck Surgery, Zhongda Hospital, Southeast University, Nanjing, China; ^4^Department of Radiology, Zhongda Hospital, Southeast University, Nanjing, China

**Keywords:** resting-state functional magnetic resonance imaging, obstructive sleep apnea–hypopnea syndrome, voxel-mirrored homotopic connectivity, functional connectivity, cognitive deficits

## Abstract

**Objective:**

To determine the changes in interhemispheric functional coordination in patients with obstructive sleep apnea–hypopnea syndrome (OSAHS) relative to controls, using a recently introduced method of analysis: voxel-mirrored homotopic connectivity (VMHC).

**Methods:**

Twenty-nine patients with OSAHS and twenty-six normal sex-, age-, and education-matched controls were recruited and resting-state functional magnetic resonance imaging data were obtained. We employed VMHC to analyze the interhemispheric functional connectivity differences between groups. The *z*-values of alterations in VMHC in brain region were correlated with clinical characteristics.

**Results:**

Compared with controls, patients with OSAHS had significantly higher scores for body mass index (*t* = 5.749, *P* < 0.001), apnea–hypopnea index (AHI; *t* = 7.706, *P* < 0.001), oxygen desaturation index (*t* = 6.041, *P* < 0.001), and Epworth sleepiness scale (*t* = 3.711, *P* < 0.001), but significantly lower scores on the Rey–Osterrieth complex figure test-immediate recall (*t* = −3.727, *P* < 0.05). On the same basis, the VMHC showed significant increases in bilateral calcarine cortex and precuneus. Moreover, significant, positive correlations were found in only these areas between the AHI and the VMHC change coefficients (*r* = 0.399, *P* = 0.032; *r* = 0.378, *P* = 0.043).

**Conclusion:**

We found a memory defect in patients with OSAHS. The correlation between the abnormal VMHC and the AHI in patients with OSAHS suggested that AHI might be a key factor in cognitive dysfunction, which might offer new insights into the neural pathophysiology underlying OSAHS-related cognitive deficits.

## Introduction

Obstructive sleep apnea–hypopnea syndrome (OSAHS) is a common, chronic sleep disease, whose characteristics include repeated episodes of partial or complete obstruction of the upper airway, and continual diaphragmatic efforts to breathe during sleep (1). Demographic studies show that the incidence of obstructive sleep apnea for middle-aged people is 2–4%, making it a major public health problem (2). Several cognitive domains could be affected by OSAHS, resulting in psychomotor dysfunction, memory weakness, decrements in vigilance and attention, and executive dysfunctions (3–5). Over the last 20 years, it has been shown that OSAHS can produce functional and structural alterations in the brain (6–8). Several studies using a voxel-based morphometry method found gray matter loss in patients with OSAHS compared with healthy controls in multiple brain regions, including posterior lateral parietal cortex, inferior temporal gyrus, hippocampus, parahippocampal gyrus, anterior cingulate gyrus, quadrangular lobule, and cerebellum (9–11). Using diffusion tensor imaging-based mean diffusivity procedures, global brain mean diffusivity values were found to be significantly decreased in patients with OSAHS. Some brain regions are especially affected, which may be a result of axonal, glial, and other cell alterations (12). In arterial spin labeling imaging, patients with OSAHS showed decreased cerebral blood flow values in multiple bilateral brain regions, including superior cerebellar peduncle, corticospinal tract, and pontocerebellar tract (13). Magnetic resonance spectroscopy has proved to be a useful neuroimaging tool able to detect alterations in cerebral metabolism, which may reflect pathological insults to brain integrity. Several magnetic resonance spectroscopy studies have revealed remarkable metabolic alterations in OSAHS (14–16).

Resting-state functional magnetic resonance imaging (MRI) based on blood-oxygenation-level dependent contrast (perhaps better known as BOLD contrast) has been widely used in various functional connectivity (FC) studies ranging from examinations of psychiatric disorders to neurological conditions (17), as well as human brain function studies (18–20). Previous neuroimaging studies have demonstrated altered brain function and connectivity in patients with OSAHS. The main findings are as follows: (1) significantly reduced FC within the anterior default mode network (DMN), bilateral fronto-parietal network, and sensorimotor network but increased FC within the posterior DMN (21), (2) the FC within the DMN, especially between the insula and the other DMN regions, is disrupted, and this abnormal FC associates with the severity of OSAHS (22, 23), and (3) resting-state FC disturbances of DMN are present in patients with OSAHS, as revealed by CONN software in our previous study (24).

As the largest commissural fiber bundle, the corpus callosum can facilitate communication and integration of emotional, cognitive, motor, and sensory information between the two cerebral hemispheres (25, 26). Despite its importance, little information is available regarding alterations in interhemispheric FC in patients with OSAHS. Diffusion tensor imaging has demonstrated changes in white matter integrity in the corpus callosum of these patients (27). Pathological alterations of the corpus callosum could impair the interhemispheric functional interactions that are foundational for the integration of executive control and attentional processing (28, 29). This is consistent with the observation of dysfunctions of executive control and attention in patients with OSAHS (3–5). In general, these studies raise the question of whether there are significant differences in interhemispheric functional coordination between patients with OSAHS and normal controls.

To explore this issue, we used calculations of voxel-mirrored homotopic connectivity (VMHC) to investigate interhemispheric FC. This method is a voxel-wise measurement of functional homotopy that reveals the synchrony of resting-state BOLD fluctuations between a voxel in one hemisphere and its mirror-image counterpart in the other. VMHC has been successfully applied to investigate interhemispheric functional homotopy in several diseases including autism, cocaine addiction, and schizophrenia (30–34). VMHC is an emerging method of analyzing the resting-state, functional magnetic resonance image (rs-fMRI). As far as we know, ours is the first study to use it to explore interhemispheric synchrony and thus to obtain correlations between measures of interhemispheric FC and clinical variables in OSAHS.

## Materials and Methods

### Subjects

Twenty-nine newly diagnosed, untreated patients with OSAHS and twenty-six normal gender-, education-, and age-comparable controls were recruited by the Sleep Laboratory of the Otolaryngology-Head and Neck Surgery Department of Zhongda Hospital, Southeast University. No patient had comorbid conditions such as heart failure or central nervous system diseases (e.g., epilepsy, stroke, and tumor) or a history of psychotropic drug use. Patients with a history of airway, laryngeal, or pharyngeal surgery were also excluded. All controls were healthy, without any history of brain abnormalities or heart failure. All participants in both the OSAHS and control groups were between 20 and 60 years of age, right-handed, and free of metallic implants or other contraindications for MRI scan.

This study was carried out in accordance with the recommendations of the Institutional Ethics Committee of Zhongda Hospital, Southeast University, Nanjing. Before the study, all participants gave written, informed consent in accordance with the Declaration of Helsinki and the protocol was approved by the Institutional Ethics Committee of Zhongda Hospital, Southeast University, Nanjing.

### Cognition and Sleep Assessment

We used the mini-mental state examination (MMSE) to screen for dementia and cognitive impairments (35). The Rey–Osterrieth complex figure test (CFT) and the logical memory test (LMT) of the Wechsler memory scale-revised were used for evaluating memory, attention, and executive functions (36). The Epworth sleepiness scale (ESS) was applied to evaluate sleep quality and daytime sleepiness (37).

### Polysomnography

The night before functional MRI (fMRI) scanning, the OSAHS and control groups were asked to undergo polysomnography monitoring (YH-2000A system) in the Department of Otolaryngology-Head and Neck Surgery of Zhongda hospital. All participants were prohibited from drinking alcohol or caffeinated beverages for 12 h before polysomnography. For all participants, polysomnography was performed from 11:00 p.m. to 07:00 a.m. A standard encephalogram (nine channels, used to distinguish between sleep and wakefulness, sleep stages, and to measure the proportion of sleep stages), chin electromyography, electrocardiography, oculography, oral cavity and nasal cavity airflow, movements of the chest and abdomen, oximeter, physical position, and stertor were recorded. Two indexes related to sleep were then calculated in accordance with the criteria of the American Academy of Sleep Medicine (38, 39). These were apnea–hypopnea index (AHI, total number of hypopnea and apnea events per hour of sleep) and oxygen desaturation index (ODI, the number of desaturation events per hour of sleep). A hypopnea was defined as a drop in breathing extent of 30% lasting at least 10 s, associated with repeated respiratory effort and a drop in oxygen saturation of at least 4%. An obstructive apnea was defined as a drop in respiratory amplitude of 90% lasting at least 10 s, associated with continued or increased inspiratory effort. A desaturation was defined as a drop in oxygen saturation of at least four percent lasting at least 3 s.

### Magnetic Resonance Imaging

Functional and structural MRI scans were performed on all participants at the Radiology Department of Zhongda Hospital using a 3.0 T MRI scanner (MAGENETOMTrio, Siemens, Erlangen, Germany). We adopted earplugs to reduce scanning noise and foam padding to decrease head motion. All participants were asked to close their eyes and not sleep, not to think about anything in particular, and to avoid any head motion as far as possible during scanning. The rs-fMRI data were acquired axially by applying an echo-planar imaging sequence sensitive to BOLD contrast. One hundred eighty functional images were obtained covering the whole brain (repetition time 2,000 ms, echo time 25 ms, flip angle 90°, field of view 240 mm × 240 mm, matrix 64 × 64, slice number 36, slice thickness 4 mm, no gap, aggregate duration of scan 6 min and 6 s). High-spatial-resolution, T1-weighted images were also obtained from each participant by applying a magnetic prepared gradient echo sequence, yielding 176 structural images (repetition time 1,900 ms, echo time 2.48 ms, flip angle 9°, field of view 256 mm × 256 mm, matrix 256 × 256, slice thickness 1 mm, no gap, duration 4 min and 18 s). Furthermore, 20 fluid-attenuated inversion-recovery images (perhaps better known as FLAIR images) were acquired to screen for structural brain lesions (repetition time 8,500 ms, echo time 94 ms, slice thickness 5 mm, duration 1 min and 59 s). The total duration of scanning was 12 min, 23 s.

### MRI Data Processing

Functional MRI data analysis was performed using DPARSFA (rs-fMRI Advanced Edition) with statistical parametric mapping (SPM8^1^) and rs-fMRI data analysis toolkits (REST^2^) with the MATLAB 2010a platform (40). A total of 180 volumes were acquired. The first 10 volumes were discarded because of instability in the initial MRI signals and adaptation of the participants to the scanner environment, and the remaining 170 volumes were submitted to the following processing, beginning with slice timing adjustment for acquisition time delays between different slices and realignment for head motion correction. (Any participant whose head motions were more than 2.0-mm of translation or more than 2.0° of rotation was excluded.) Then the fMRI data were spatially normalized to the Montreal Neurological Institute template with resampling to a 3 mm × 3 mm × 3 mm voxel size, smoothing with an isotropic Gaussian kernel (full-width at half maximum = 4 mm) and finally, detrending and bandpass filtering at 0.01–0.08 Hz.

### VMHC Analysis

We used REST software to compute the VMHC. The Pearson’s correlation coefficient between the remaining time series of each voxel and that of its mirrored interhemispheric voxel was calculated and the homotopic FC was measured (41). Subsequently, the correlation values were converted to *z*-values using Fisher’s *r*-to-*z* transformation to enhance the stability of the values. The resulting values were considered a measure of VMHC and were used for the final intergroup analysis.

### Statistical Analysis

A two-sample *t*-test was used for VMHC-map analysis to investigate altered interhemispheric FC in patients with OSAHS. *P* < 0.05 was considered statistically significant, and cluster sizes were set at 63 voxels, as determined by Monte Carlo simulations using the AFNI AlphaSim program. Because of the influence of micromovements from volume to volume on the FC (42), framewise displacement values were calculated to reflect the temporal derivative of the movement parameters for each patient. Analysis revealed no remarkable difference in the mean framewise displacement values between patients with OSAHS and normal controls. Finally, a Pearson correlation analysis was performed to explore the correlations between VMHC and clinical variables relevant to OSAHS including AHI, ODI, body mass index (BMI), MMSE, CFT, LMT, and ESS. Partial correlation with some covariates (age, sex, and years of education) was performed to evaluate the effects of these covariates on increased VMHC.

Two-sample *t*-tests and χ^2^-tests were used to analyze differences in demographic data between patients with OSAHS and normal controls. *P* < 0.05 was considered to indicate statistical significance.

## Results

### Demographic and Clinical Data

In our study, 55 participants (29 patients with OSAHS and 26 normal controls) were included in the final data analysis. The demographic and clinical data of all participants are presented in Table 1. No significant differences were found between patients with OSAHS and controls in age, sex, or years of education (*P* > 0.05). However, the patients with OSAHS had significantly higher scores for BMI (*t* = 5.749, *P* < 0.001), AHI (*t* = 7.706, *P* < 0.001), ODI (*t* = 6.041, *P* < 0.001), and ESS (*t* = 3.711, *P* < 0.001), but significantly lower scores for CFT-immediate recall (*t* = −3.727, *P* < 0.05) compared with controls. No significant differences were observed between patients with OSAHS and controls in CFT-delayed recall (*t* = −1.862, *P* > 0.05), LMT-immediate recall (*t* = −0.322, *P* > 0.05), LMT-delay recall (*t* = −0.371, *P* > 0.05), or score on the MMSE (*t* = −1.439, *P* > 0.05).

**Table 1:** Population statistics and clinical information.

Characteristics	Controls (26)	OSAHS (29)	*P* value	*t* Value	ES
Age	34.46 ± 9.97	39.62 ± 9.95	0.061	1.918	0.250
Sex (female/male)	8/18	6/23	0.392	0.734	0.592
Education (years)	13.96 ± 2.58	12.67 ± 3.17	0.103	−1.658	−0.218
BMI	22.16 ± 2.93	27.99 ± 4.37	0.000	5.749	0.617
AHI	2.43 ± 1.68	33.67 ± 21.75	0.000	7.706	0.711
ODI	0.93 ± 1.03	32.07 ± 27.73	0.000	6.041	0.621
MMSE	28.96 ± 1.51	28.34 ± 1.65	0.156	−1.439	−0.192
CFT-immediate recall	35.92 ± 0.39	35.17 ± 1.00	0.001	−3.727	−0.442
CFT-delay recall	21.88 ± 5.96	18.48 ± 7.41	0.068	−1.862	−0.245
LMT-immediate recall	20.69 ± 6.19	20.07 ± 7.93	0.749	−0.322	−0.043
LMT-delay recall	19.77 ± 5.88	19.03 ± 8.41	0.712	−0.371	−0.051
ESS	4.81 ± 2.61	7.69 ± 3.09	0.000	3.711	0.449

*Averages are accompanied by the 95% confidence limits*.

*OSAHS, obstructive sleep apnea–hypopnea syndrome; ES, effect size; BMI, body mass index; AHI, apnea–hypopnea index; ODI, oxygen desaturation index; MMSE, mini-mental state examination; CFT, Rey–Osterrieth complex figure test; LMT, logical memory test of the Wechsler memory scale-revised; ESS, Epworth sleepiness scale*.

*For the sex ratio, the χ^2^ value substitutes for the t-value*.

### Changes in VMHC in Patients With OSAHS Compared With Normal Controls

**Figure 1:**
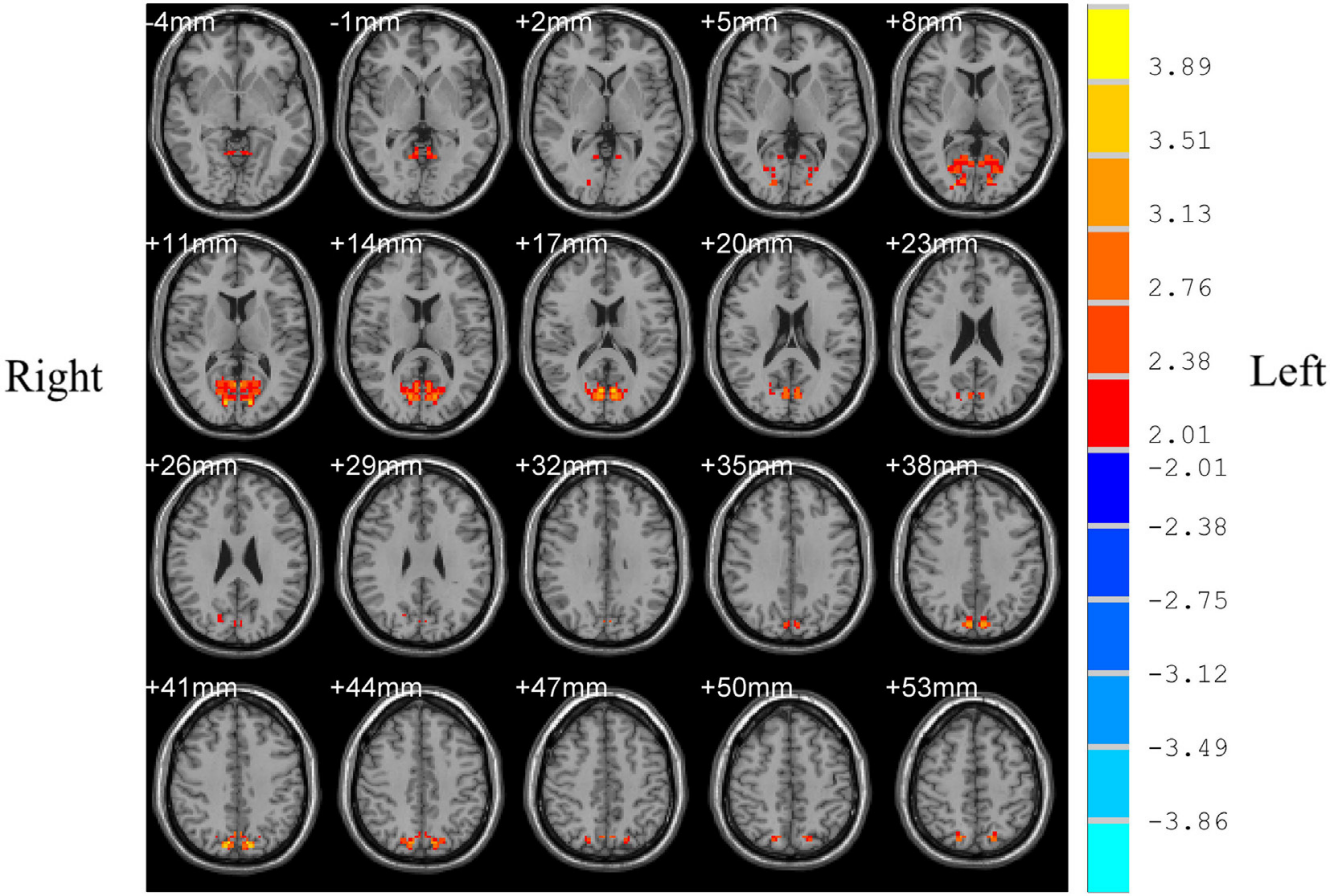
Shown is a significantly increased voxel-mirrored homotopic connectivity (VMHC) in obstructive sleep apnea–hypopnea syndrome patients as compared with controls. Thresholds are set at a corrected *P* < 0.05, as determined by Monte Carlo simulation. Red indicates increased VMHC. The left side of the images represents the right of the brain and *vice versa*.

**Figure 2:**
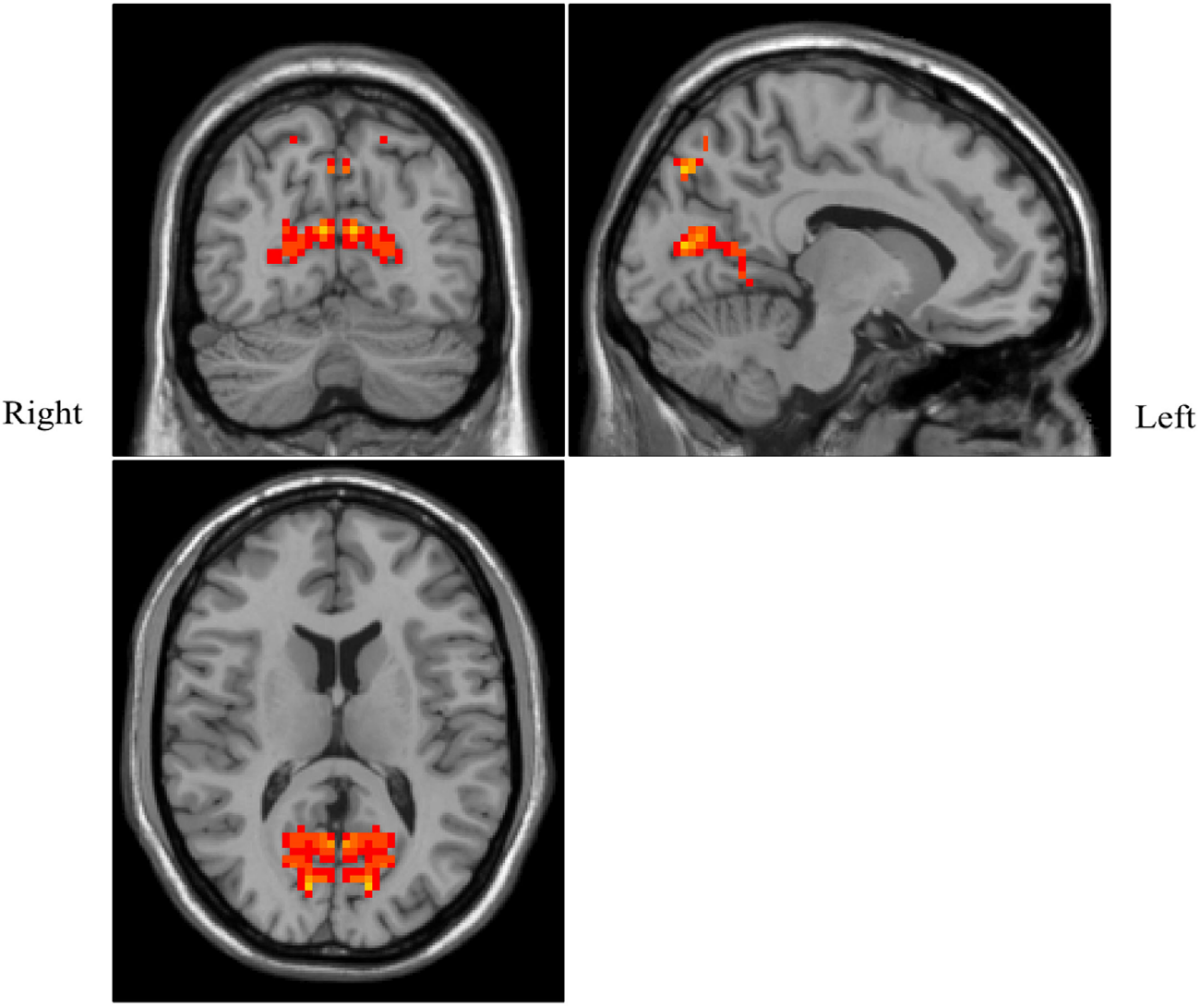
Shown is a significantly increased voxel-mirrored homotopic connectivity (VMHC) in obstructive sleep apnea–hypopnea syndrome patients as compared with controls. Thresholds are set at a corrected *P* < 0.05, as determined by Monte Carlo simulation. Red indicates increased VMHC. The left side of the images represents the right of the brain and *vice versa*.

Patients with OSAHS showed significantly increased VMHCs in the calcarine cortex and precuneus compared with healthy controls. However, relatively decreased VMHCs were not seen in patients with OSAHS. The details are presented in Figures 1 and 2 and Table 2.

**Table 2:** Brain areas with differences in voxel-mirrored homotopic connectivity between OSAHS patients and controls.

Brain area	BA	MNI	*t* Value	Voxels
Precuneus	7	±9, −78, 42	4.023	63
Calcarine	18	±6, −69, 18	3.545	198

*BA, Brodmann area; MNI, Montreal Neurological Institute coordinates; OSAHS, obstructive sleep apnea–hypopnea syndrome*.

### Correlation Analysis

**Figure 3:**
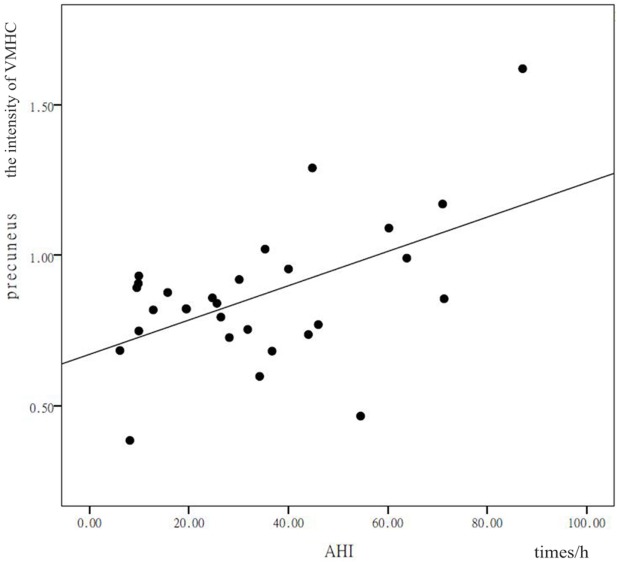
Shown is a significant positive correlation between the voxel-mirrored homotopic connectivity (VMHC) values in the precuneus and calcarine cortex, and the apnea–hypopnea index (AHI). *X* represents the AHI and *Y* represents the intensity of the VMHC.

**Figure 4:**
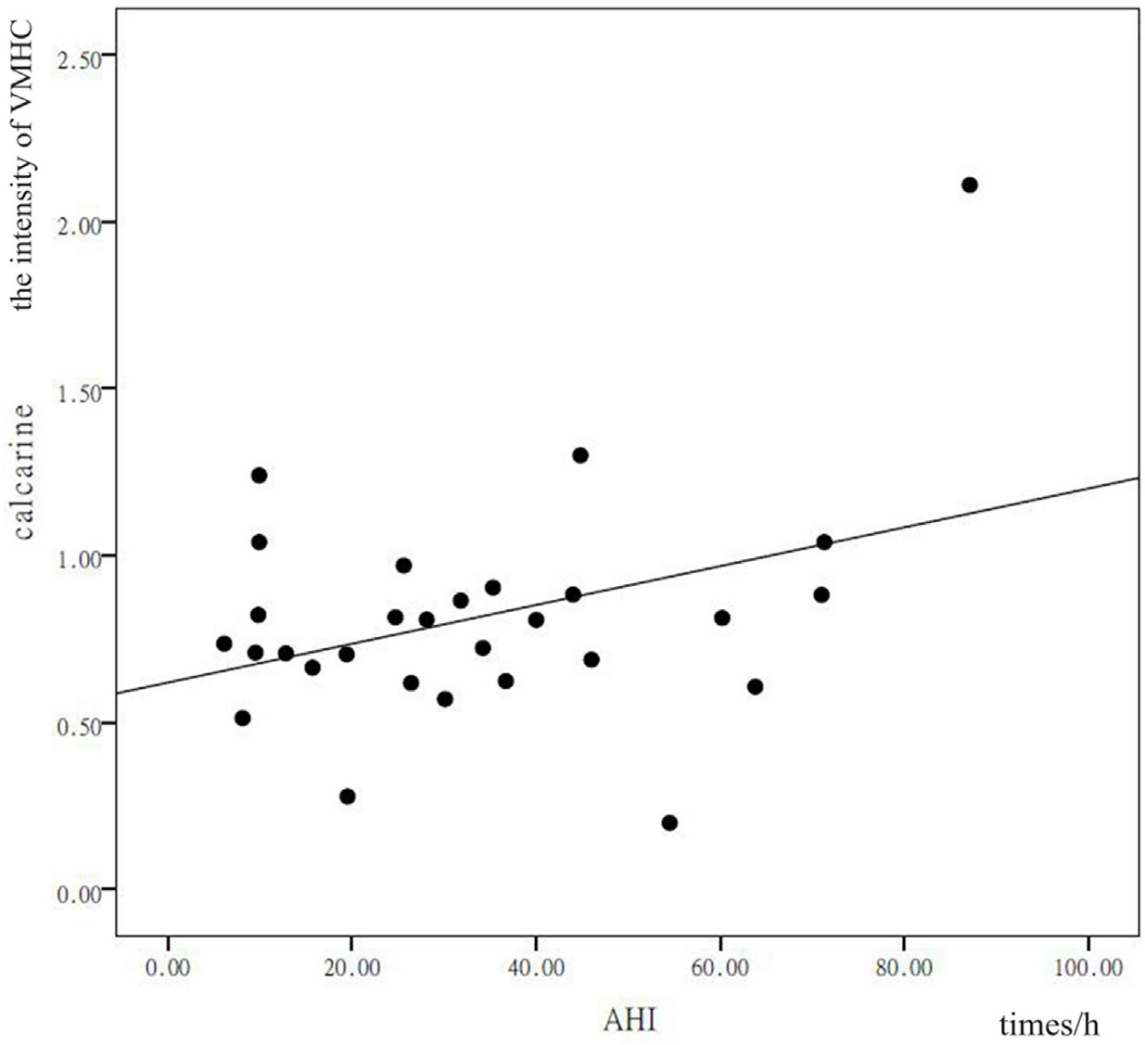
Shown is a significant positive correlation between the voxel-mirrored homotopic connectivity (VMHC) values in the precuneus and calcarine cortex, and the apnea–hypopnea index (AHI). *X* represents the AHI and *Y* represents the intensity of the VMHC.

In patients with OSAHS, a significant positive correlation was found in the precuneus between VMHC values and AHI (*r* = 0.378, *P* = 0.043). In addition, a significant positive correlation was found in the calcarine cortex between VMHC values and AHI (*r* = 0.399, *P* = 0.032) (see Figures 3 and 4). VMHC did not associate with ODI, BMI, MMSE, CFT, or ESS. There were no significant correlations between increased VMHC and any covariate (age, *P* = 0.676, *r* = 0.106; gender, *P* = 0.433, *r* = 0.197; years of education, *P* = 0.636, *r* = −0.120).

## Discussion

In this study, we adopt a VMHC approach to investigate possible differences in interhemispheric FC between patients with OSAHS and controls. We found a significant increased VMHC in the precuneus and calcarine cortex in patients with OSAHS compared with controls. What is more, there were significant positive correlations between the AHI and the VMHC in these brain areas.

Few past studies have reported increased activities in the precuneus in patients with OSAHS. However, here we found significantly increased VMHC in the precuneus of such patients. Huynh and coworkers found no difference in gray matter volume between patients with moderate-to-severe OSAHS and normal controls, so an increased VMHC was not attributed to structural differences, but possibly to strengthened neural activity (43). Our findings are consistent with a previous report of an abnormal overactivation in the precuneus of OSAHS patients. That report suggested that the overactivation represents an adaptive compensatory response (44).

The precuneus is part of the posterior DMN, which plays an important role in fundamental cognitive functioning, including episodic memory retrieval, visuo-spatial imagery, self-processing, and consciousness (45, 46). The DMN involves a set of brain regions that are more active during rest than during goal-directed tasks and is involved in a wide range of higher-order cognitive functions (47). Several systematic meta-analytic studies have highlighted the importance of the DMN in cognition and clinical symptoms (6, 48). It has been reported that abnormal activity in the DMN, such as abnormal resting-state FC among its sub regions, significant regional deficits in spontaneous activity, and a decrease of blood-oxygenation-dependent fluctuations in its main nodes, correlate with sleep parameters and delay memory (49–51). These findings imply the presence of cognitive deficits in OSAHS patients. Similarly, the altered VMHC in the precuneus demonstrated here implies a dysfunction of the DMN, which might suggest the presence of cognitive impairments in the OSAHS patients of our study.

Apart from any cognitive deficits that can arise from these abnormalities, these changes imply problems in sustained and divided attention that arguably contribute to the well-recognized driving difficulties of patients with OSAHS (52) and may also lead to dysfunctional sleep rhythms (53). Moreover, we also found a positive correlation between enhanced VMHC and the AHI in patients with OSAHS. It has long been speculated that repeated hypopnea leads to aberrant properties in the DMN and precuneus, resulting from an adaptive compensatory response tending to maintain the normal activities of the brain.

The calcarine cortex is a main core of the visual recognition network (54), and has been associated with attentional shifts to an expected visual goal and with modulation of visual input by attention, especially when visual information is used to guide saccades or reaching (55). Previous neuroimaging studies have shown that patients with OSAHS have impairment in several cognitive domains, including executive control function, episodic memory, coordination of movement, and attention (3–5). However, only a few studies have reported visual dysfunction in patients with OSAHS. Giora and coworkers found that in a visual task, patients with OSAHS had a significantly longer reaction times than controls, indicating the presence in these patients of an impairment involving basic mechanisms of visual processing (56). Another study demonstrated that retinal nerve-fiber thickness is reduced in patients with OSAHS (57). It has also been reported that OSAHS leads to a high prevalence of open-angle glaucoma and visual-field defects (58, 59). In this study, the aberrant VMHC found in the calcarine cortex in the patients with OSAHS may indicate visual dysfunction. Furthermore, Chan and coworkers found impaired attention and visual–fine-motor coordination in children with OSAHS (60). Other research found an increased risk for traffic accidents in patients with OSAHS (61). We thus speculate that disorders of coordination of vision and motion might result in patients with OSAHS having an extra risk of positioning dislocations and accidents. In addition, since VMHC in the calcarine cortex here had a positive correlation with AHI, we suggest that AHI might be a key factor in visual dysfunction and that the calcarine area is sensitive to the effects of apnea and hypopnea.

Recently, a systematic meta-analysis of patients with OSAHS demonstrated co-activations in several bilateral brain regions including amygdala, hippocampus, thalamus, precuneus, and posterior cingulate cortex. This suggested a significant association of the disorder with affective and emotional processing, as well as with memory-related processes (62). Similarly, our results are largely in agreement with the above-mentioned findings, and it also follows that the dysfunctions of memory, attention, and executive control reported in patients with OSAHS might be partly explained by increased interhemispheric functional interactions in precuneus and calcarine cortex. Moreover, patients with OSAHS have an increased prevalence of several psychiatric disorders including major depressive disorder and anxiety (63). Studies have also found aberrant interhemispheric FC in patients with depression (64, 65). We speculate that malfunctions within interhemispheric FC could conceivably contribute to the genesis of the neuropsychiatric deficits previously reported to be prevalent in OSAHS, such as depression, emotional lability, and anxiety.

## Conclusion

Interhemispheric functional interactions are at the foundation of the integration of executive control and attentional processing (61). Our study found increased homotopic connectivity (VMHC) in bilateral precuneus and calcarine cortex in patients with OSAHS. Moreover, we found positive correlations between enhanced VMHC in precuneus and calcarine cortex and the AHI. These results indicate that abnormal VMHC may provide an early biomarker for the detection of cognitive impairments in patients with OSAHS, advantageously using a noninvasive imaging technology. However, several limitations of this study should be noted. First, the study should be repeated with a larger sample size to eliminate any possible instability in the results. Second, some participants were unable to accommodate an encephalogram examination, so the encephalogram data in our study is incomplete. In the future, we will endeavor to obtain complete encephalogram data and examine the rapid-eye-movement-specific features of OSAHS. Third, we focused here on cognitive changes occurring in mixed-severity patients with OSAHS. A study specific to the mild-to-severe group should be conducted, which would offer powerful insights into neurophysiological mechanisms in patients with a distinct level of OSAHS. Finally, longitudinal studies are needed to provide imaging markers for clinical treatment.

## Ethics Statement

All participants provided written informed consent before the study, and the protocol was approved by the institutional Ethics Committee of Zhongda Hospital, Southeast University, Nanjing.

## Author Contributions

MY, BL, and Z-CH contributed conception and design of the study; Y-TL, H-XZ, TC, and LZ organized the database; Y-TL performed the statistical analysis; Y-TL wrote the first draft of the manuscript; H-JL and Y-QH wrote sections of the manuscript. All authors contributed to manuscript revision, read and approved the submitted version.

## Conflict of Interest Statement

The authors declare that the research was conducted in the absence of any commercial or financial relationships that could be construed as a potential conflict of interest.

## Footnotes

^1^http://www.fil.ion.ucl.ac.uk/spm/ (Accessed: 2010).

^2^http://www.restfmri.net/ (Accessed: 2011).

## Abbreviations

AHI, apnea–hypopnea index; CFT, Rey–Osterrieth complex figure test; DMN, default mode network; ESS, Epworth sleepiness scale; FC, functional connectivity; LMT, logical memory test; MMSE, mini-mental state examination; MRI, magnetic resonance imaging; ODI, oxygen desaturation index; OSAHS, obstructive sleep apnea–hypopnea syndrome; VMHC, voxel-mirrored homotopic connectivity.
